# Comparison of Donkey, Pony, and Horse Dorsal Profiles and Head Shapes Using Geometric Morphometrics

**DOI:** 10.3390/ani12070931

**Published:** 2022-04-05

**Authors:** Małgorzata Maśko, Małgorzata Wierzbicka, Łukasz Zdrojkowski, Tomasz Jasiński, Urszula Sikorska, Bartosz Pawliński, Małgorzata Domino

**Affiliations:** 1 Department of Animal Breeding, Institute of Animal Science, Warsaw University of Life Sciences (WULS–SGGW), 02-787 Warsaw, Poland; malgorzata_masko@sggw.edu.pl (M.M.); s193600@sggw.edu.pl (U.S.); 2 Department of Large Animal Diseases and Clinic, Institute of Veterinary Medicine, Warsaw University of Life Sciences (WULS–SGGW), 02-787 Warsaw, Poland; malgorzata_wierzbicka@sggw.edu.pl (M.W.); tomasz_jasinski@sggw.edu.pl (T.J.); bartosz_pawlinski@sggw.edu.pl (B.P.)

**Keywords:** posture, skull, size, landmarks, equids

## Abstract

**Simple Summary:**

With the increasing interest toward donkey breeding and equid welfare, the scales and methods of welfare assessment, which were successfully designed and validated for horses, are a starting point for the development of similar approaches for donkeys. As horses and donkeys are morphometrically different, the current study aimed to compare donkey, pony, and horse dorsal profiles and head shapes. Geometric morphometrics (GM) was applied to characterize the shapes and sizes of the studied equids based on the analysis of the photographs of 14 donkeys, 14 ponies, and 14 horses. The donkeys differed from ponies and horses in the shape of the dorsal profile and the head shape, but only from horses in the size of the evaluated centroids. Moreover, the ponies and horses differed in size but not in the shape of the investigated lines reflecting the posture and head contour. When both lines were compared, the GM distances (the Mahalanobis and Procrustes distances) were higher between donkeys and ponies and horses than between ponies and horses. GM revealed the differences in dorsal profiles and head shapes between equids, which should be considered when adapting scales and methods of welfare assessment from horses to donkeys.

**Abstract:**

Since donkey breeding has increased due to their variety of uses, welfare evaluation has become more important. This study aimed to compare donkey, pony, and horse dorsal profiles and head shapes using geometric morphometrics (GM). Photographs of 14 donkeys, 14 ponies, and 14 horses were analyzed using GM, including the sliding semilandmarks method. The variations in the first three principal components (PCs) were PC1: 57.16%, PC2: 16.05%, and PC3: 8.31% for the dorsal profiles and PC1: 44.77%, PC2: 13.46%, and PC3: 7.66% for the head shapes. Both the dorsal profiles and head shapes differed between donkeys and horses (*p* < 0.0001) but not between donkeys and ponies (*p* > 0.05). Moreover, both the dorsal profiles and head shapes differed in size between ponies and horses (*p* < 0.0001) but not in shape (*p* > 0.05). Higher Mahalanobis and Procrustes distances were noted between donkeys and horses as well between donkeys and ponies than between ponies and horses. The use of geometric morphometrics revealed the differences in the dorsal profiles and head shapes between the studied equids. These differences should be taken into account when adapting welfare scales and methods from horses to donkeys.

## 1. Introduction

The global donkey population has been steadily rising since 1961 [1], and this trend is set to continue [2]. Donkeys are engaged in a wide variety of working [3,4,5] and non-working [6,7,8,9,10,11,12] roles, with the latter including meat and milk production [6,7] and uses in nutraceuticals [8], cosmetics [9], silviculture [10], tourism [11], and onotherapy [12], which are becoming more popular. The increasing interest in these equids’ roles has been accompanied by the conviction that healthier working animals can work more efficiently than those under poor conditions [13,14]. Therefore, as improving the welfare of working equids also provides benefits for their owners [2], the scales and methods of welfare assessment, which were successfully designed and validated for horses, should be adapted to welfare evaluation in donkeys [15].

Due to the increased interest and popularity of donkeys, the largest welfare and management concerns for donkeys kept as companion, guard, and pleasure animals are obesity and laminitis [16,17,18]. Many donkeys housed under conditions typical for horses are overweight. Donkeys are certainly less demanding, as they can feed on poor-quality forages [16,19]. Therefore, the monitoring of the nutritional status of donkeys is one of the most important welfare indicators characteristic of this species. In donkeys, a five-point scale of the body condition score (BCS) [16], the fatty neck score (FNS) [19], and the dental condition score (DCS) [19] were proposed as most effective methods for determining the overall nutritional status and welfare [17]. Recently, both BCS and FNS have been shown as factors affecting the dorsal profile of donkeys [20]. Donkeys’ body condition should be considered when their posture is investigated in relation to chronic stress [21,22] or depression-like syndrome [23,24]. Horse posture and the shape of the dorsal profile were successfully explored using z geometric morphometrics (GM) and described as effective indicators of the welfare state [23,24]. Thus, in some donkey-specific conditions, the use of GM as the welfare assessment method can be considered useful and valid for donkeys [20].

The second problem with companion, guard, and pleasure donkeys is their typical nature and temperament, which makes detection of pain or signs of colic and laminitis challenging for owners [17,18,25]. The temperament in donkeys differs from that in horses, as donkeys are generally less overtly reactive, demonstrating a more stoic nature [15]. While generally regarded as a flight response animal, donkeys demonstrate frightening behavior much more rarely than horses, rather freezing in response to threatening stimuli [26]. Therefore, donkeys often hide pain [15,26], exhibiting more subtle signs of pain [15]. Recently, every effort was made to adopt the horse scales of pain to detect its more subtle signs in the donkeys [15,27] in order to improve previously unscaled pain assessments in donkeys [28,29,30,31]. From a few valuable scales of horse pain assessment [32,33,34,35,36], the facial expression-based pain scale [34,35,36] proved to be a valid and clinically applicable pain scale for donkeys with different types of acute pain [15]. A facial expression analysis was also proposed in a hierarchical model for pain estimation in equids, which aimed at pose-specific automatic pain prediction [27]. The automatic facial landmarking and pain estimations were explored using histogram of oriented gradients features with a support vector classifier, receiving a high score on pose and pain estimation for horses but not for donkeys. The difficulties of transferring horse models to donkeys’ faces have been shown in the case of automatic landmarking and pain estimation [27]. As neither facial expression-based acute pain assessments [15] nor automatic pain detection methods [27] are directly transferable from horses to donkeys, the differences in head morphology should also be considered. As shown in the post-mortem evaluation, donkeys have smaller and shorter heads than horses. Specific features are their lower skull weight, smaller cranial width, smaller mandibular depth, and shorter cranial length [37]. In the research by Merkies et al. [37], no differences were found in donkey nasal length [37], which is in contradiction to the theory by Hummel et al. [27], whereby differences in face proportions caused by distinct nose lengths cause difficulties in the implementation of automatic pain detection [27].

There is a clear need for morphometric interspecies comparisons to facilitate future research on the transfer of scales [15,27] and methods [20,23,24,27] used in welfare assessments from horses to donkeys. As donkeys range in size much more than horses [37], a high index of individual volatility is predicted. Thus, a reliable and objective correction of geometric definitions of donkey size and shape would be a major step forward for enabling early intervention in posture and pain problems, improving donkey welfare. We hypothesize that horses and donkeys are morphometrically different, which may affect the results of dorsal line and facial-expression-based pain evaluations, and which may be evidenced on live animals in field conditions. Therefore, the current study aimed to compare donkey, pony, and horse dorsal profiles and head shapes using geometric morphometrics.

## 2. Materials and Methods

### 2.1. Animals

The study was conducted on healthy donkeys (n = 14), ponies (n = 14), and full-size horses (n = 14). The donkey group included seven females and seven males with a mean age ± SD of 8.93 ± 2.30 and with a mean height at withers ± SD of 122.93 ± 10.16. Donkeys were mixed breeds, such as Andalusian (n = 2), Grigio Siciliano (n = 2), Martina Franca (n = 5), Magyar Parlagi (n = 2), and Romanian (n = 3) breeds mixed with the local mixed-breed donkeys. The pony group included eight mares and six geldings with a mean age ± SD of 8.57 ± 3.13 and with a mean height at withers ± SD of 140.57 ± 2.53. Ponies were predominantly a native Polish pony breed, Konik Polski (n = 11), as well two Haflingers (n = 2) and one Connemara (n = 1). The horse group included six mares and seven geldings with a mean age ± SD of 9.71 ± 3.17 and with a mean height at withers ± SD of 166.79 ± 3.53. Horses were predominantly Polish warmblood breeds, including a Polish halfbred horse (n = 9), Wielkopolska (n = 3), and Malopolska (n = 1), whereas one horse was thoroughbred (n = 1).

The donkeys were privately owned and were kept under the same conditions, in the same stable located in southern Poland in Lubachów. All animals were fed three times a day with a personalized dose of meadow hay, with access to water ad libitum, and had daily access to grass pastures for no shorter than 8 h per day. Animals did not work and were housed as companion animals. The ponies and horses were WULS (Warsaw University of Life Sciences)-owned and were housed with the same management in the Didactic Stable of Horse Breeding Division and the Stable of Large Animals Disease and Clinic Division at WULS. Ponies and horses received an individually calculated ration of hay, oats, and concentrate according to nutritional requirements arising from maintenance and workload and were given water ad libitum. All ponies and horses were in daily leisure use in the riding school, ridden 1–2 h a day, 5 days a week, and had daily access to sandy paddocks for no shorter than 6 h per day.

### 2.2. Inclusion Criteria

Only completely healthy animals were included in the study. A basic clinical examination was conducted as the standard veterinary diagnostic procedure. The internal temperature, heart rate, respiratory rate, mucous membranes, capillary refill time, and lymph nodes were evaluated following international veterinary standards [38]. A detailed examination of the musculoskeletal system was performed following the guidelines for the lameness evaluation of an athletic horse [39]. None of the donkeys, ponies, or horses were excluded.

Since BCS and FNS were reposted to affect the dorsal line of donkeys [20], only animals that received 2 or 3 BCS in a 5-point scale of the previously described scoring system [16] and only donkeys that received 2 of 3 FNS in a 5-point scale of the previously described scoring system [19] were included in the study. The BCS was established for donkeys, ponies, and horses. Four independent researchers rated the BCS on a scale of 1 (poor) to 5 (obese) after palpation and a visual assessment of the animals. For further analysis, the median of the 4 scores rounded to the nearest whole- or half-score increment was used. None of the ponies or horses were excluded, whereas the fourteen donkeys that met the BCS-dependent criterion were selected from a group of forty total donkeys. The FNS was established only for donkeys. Four independent researchers rated the FNS on a scale of 1 (poor) to 5 (obese) after palpation and a visual assessment of the animals. For further analysis, the median of the 4 scores rounded to the nearest whole- or half-score increment was used. The fourteen donkeys that met the FNS-dependent criterion were selected from the group of forty total donkeys. Measurements taken from animals were a part of standard veterinary diagnostic procedures and did not require ethical approval, whereas photographic sessions did not require contact with the animal.

### 2.3. Geometric Morphometrics

The GM methodology was adapted from the previously described measuring system used for horse [24] and donkey [20] posture evaluations. In the current study, both the equids’ dorsal profiles and head shapes were evaluated.

**Figure 1:**
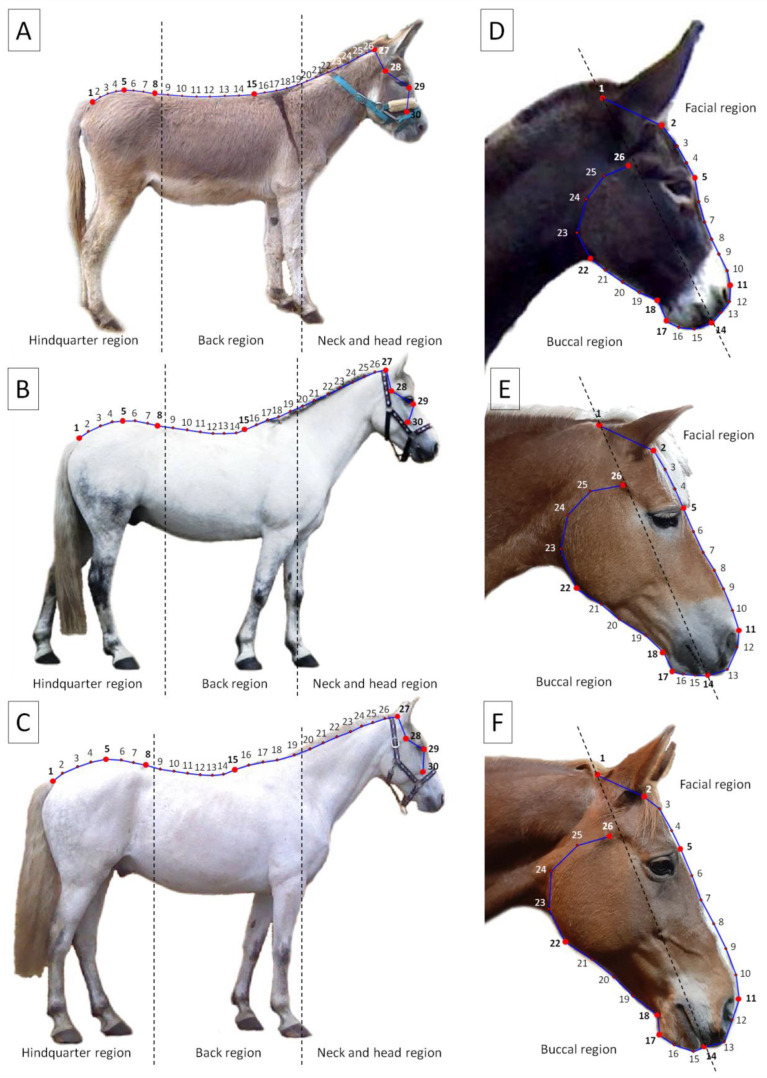
Examples of the digitalization of landmarks (LDs; marked with a big red point and bold font) and the sliding semilandmarks (SSLs; marked with a small red point) from photographs of donkeys (**A**,**D**), ponies (**B**,**E**), and horses (**C**,**F**). The blue curves were fitted to the shape of the dorsal profile (**A**–**C**) and the head (**D**–**F**). Dashed lines indicate the boundaries between regions.

For the dorsal profile evaluation, seven self-adhesive red markers (red dots) were positioned on one side of the animal’s body as seven landmarks (LDs). A medial canthus of the eye was used as the eighth LD. The side of the animal with less mane was selected. The markers were positioned in relation to easily palpable skeleton structures following recent protocols [20,23,24], as shown in Figure 1 for donkeys (Figure 1A), ponies (Figure 1B), and horses (Figure 1C), respectively. The markers were used to locate anatomical points in the photographs.

For the head shape evaluation, eight self-adhesive red markers (red dots) were positioned on one side of the animal’s body as eight LDs. The rim of the mouth was used as the ninth marker. Similarly, the side of the animal with less mane was selected. The positions of markers were adapted from Hummel et al.’s research on facial-expression-based automatic pain detection [27]. The markers were positioned around easily palpable structures, as shown in Figure 1 for donkeys (Figure 1D), ponies (Figure 1E), and horses (Figure 1F), respectively, and then used to locate anatomical points in the photographs.

#### 2.3.1. Photographs Collection

The photographs were taken outdoors with no additional lighting. An unfamiliar experimenter led an animal on a slack rope in a walk and stopped them gently to achieve spontaneous postures following previously described protocols [20,23,24]. The photographs, at 20 photos per individual, were acquired on the left or right sides at a 90° camera angle from a distance of 10 m from the animal using a Canon EOS 5D Mk2 digital camera (Canon Inc., Tokyo, Japan). The first 10 photos per individual were positioned in the center of the trunk, whereas the second 10 photos per individual were positioned in the center of the head. Out of the trunk-centered photographs, only these on which all four hooves were placed completely on the ground were included. When a hoof was off the ground or resting or when an animal would not stand still, the photographs were excluded. Out of the head-centered photographs, only those on which the animal’s head was in a true lateral position and ears were directed rostrally were included. When an animal would not stand still, the photographs were excluded. Out of a total of 840 photographs, 84 photos (per individual: 1 trunk-centered photo and 1 head-centered photo) were selected for further research based on the inclusion criteria. If required, the selected photographs were turned horizontally in order to achieve the same orientation, as the side of the animal with less mane was selected for photographing.

#### 2.3.2. Photograph Processing

The photograph processing steps were adapted from the previously described protocols [20,23,24]. The photographs saved as .JPG files were converted to .TPS files using the tpsUtil (version 2.31) software. Then, LDs reflecting the locations of the markers positioned on an animal’s body, with eight on the trunk-centered photographs and nine on the head-centered photographs, were digitized using the tpsDig2 (version 2.31) software.

The sliding semilandmarks (SSLs) method was used for the size and shape analysis to fit the curve precisely to the shapes of the dorsal profile and head, with digitized locations of the successive 22 SSLs and 17 SSLs, respectively. For the dorsal profile digitization, four curves with SSLs are marked between the first five consecutive LDs marked in Figure 1A–C as big red dots with numbers 1, 5, 8, 15, and 21. The SSLs were added to the curves by length as follows: (i) 3 SSLs between 1st and 5th LDs, (ii) 2 SSLs between 5th and 8th LDs, (iii) 6 SSLs between 8th and 15th LDs, and (iv) 11 SSLs between 15th and 21st LDs.

For the head shape digitization, six curves with SSLs are marked between the eight consecutive LDs marked in Figure 1D–F as big red dots with numbers 2, 5, 11, 14, 17, 18, 22, and 26. The SSLs were added to the curves by length as follows: (i) 2 SSLs between 2nd and 5th LDs, (ii) 5 SSLs between 5th and 11th LDs, (iii) 2 SSLs between 11th and 14th LDs, (iv) 2 SSLs between 14th and 17th LDs, (v) 3 SSLs between 18th and 22nd LDs, and (vi) 3 SSLs between 22nd and 26th LDs.

Then, the TPS curves were appended to the LDs using the tpsUtil (version 2.31) software, which allowed us to obtain 30 LDs reflecting the shape and size of the dorsal profile, as well 26 LDs reflecting the shape and size of the head.

#### 2.3.3. Photographs Analysis

The single TPS file contained ID information about the equid groups of donkeys (D), ponies (P), and horses (H). MorphoJ software version 2.0 (Copyright 2008−2019 Christian Peter Klingenberg, Licensed under the Apache License, https://morphometrics.uk/MorphoJ_guide/frameset.htm?index.htm, accessed on 20 December 2021), an integrated software package for geometric morphometrics, was used for further analyses [40]. The photographs analysis was conducted following the same protocol for the dorsal profile and head shape, independently. First, the extraction of a new classifier from ID strings was performed to classify the equid groups as D, P, and H, as well as to classify the breed groups as AD (Andalusian), GS (Grigio Siciliano), MF (Martina Franca), MP (Magyar Parlagi), RO (Romanian), KP (Konik Polski), HA (Haflinger), CO (Connemara), Polish halfbred (PH), WL (Wielkopolska), ML (Malopolska), and XX (thoroughbred). Then, the generalized Procrustes analysis (GPA, returning Procrustes coordinates), covariance matrix (CovMatrix) generation, and principal component analysis (PCA) were conducted to visualize the distribution of the shape configurations corresponding to the dorsal profile and head shape. The equid classifier variables were used to determine the color for each equid group on the general scatter plot of the principal component scores, with red for D group, blue for P group, and green for H group. The breed classifier variables were used to determine the color for each breed group on the detailed scatter plot for each equid group separately. The confidence ellipses were drawn using a 0.9 probability and a classifier as a criterion for grouping the observations. The classifier was also used to determine the colors of the ellipses and data points. The shapes of the dorsal profile and head were displayed as wireframe graphs for the first three principal components (PC) resulting from the PCA. Average observations for each equid group were executed and displayed as wireframe graphs and transformation grids.

The whole dataset was included to determine the equid (donkeys, ponies, and horses) effect on the centroid size and shape, using the Procrustes ANOVA with the significance level established as *p* < 0.05. Then, the dataset was subdivided into D, P, and H sub-datasets, which were combined into DP, DH, and PH sub-datasets. The sub-datasets were included to determine the intra-equid (DP, donkeys and ponies; DH, donkeys and horses; PH, ponies and horses) effects on the centroid size and shape, using Procrustes ANOVA with the significance level established as *p* < 0.05. The whole dataset was included to determine the distances (Mahalanobis distances, MD; Procrustes distances, PD) for the dorsal profiles and head shape among the examined categories, respectively, using the canonical variate analysis (CVA) method. At the stage of preparing figures, some LDs were grouped on the trunk-centered photographs to focus only on the hindquarter (LDs 1 to 8), back (LDs 9 to 19), or neck and head (LDs 20 to 30) regions, as well on the head-centered photographs to focus only on the facial (LDs 1 to 14) or buccal (LDs 14 to 26) regions.

## 3. Results

### 3.1. Comparison of the Equids’ Dorsal Profiles

**Figure 2:**
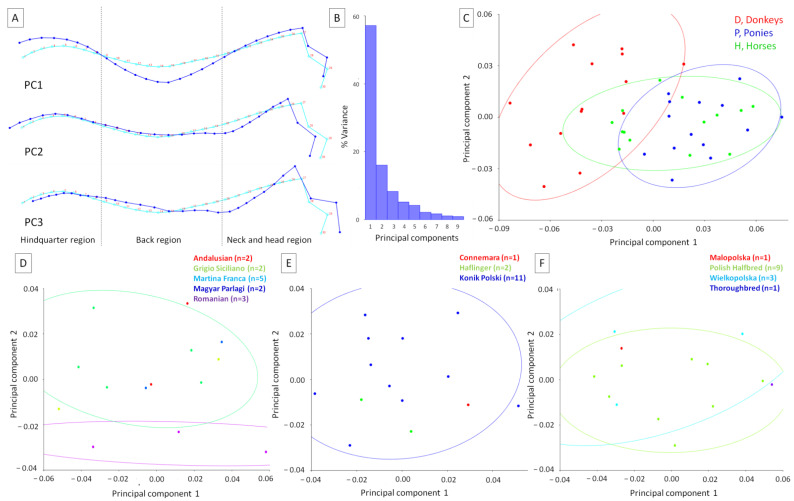
Principal components (PCs) of the dorsal profiles of equids are represented by (**A**) the wireframe graph, (**B**) the histogram of variance, and (**C**–**F**) the scatter plot of the PCs scores of the equids. On the wireframe graph (**A**), light blue landmarks and curves represent the consensus animal’s dorsal profile. Dark blue landmarks and curves represent the extremum (minimum of the axis) values for PC1, PC2, and PC3, respectively. Dashed lines indicate the boundaries between regions. For the general scatter plot of the PC scores (**C**), the color for each group was determined based on the classifier variables: D, donkeys; P, ponies; H, horses. For the detailed scatter plots of the PC scores (**D**–**F**), the color of each breed with the number of individuals (n) is shown for donkeys (**D**), ponies (**E**), and horses (**F**). The confidence ellipses were drawn using a 0.9 probability and a classifier as a criterion for grouping the observations.

The dorsal profile of the animals was first investigated to identify variations in postures among equid groups. For 30 LDs in 2 dimensions, the dataset contained 42 observations, of which 42 were included for analyses. Considering the PCA, the total variance was 0.0025 and the eigenvalue variance was 0.000000039. The eigenvalue variance scaled by total variance was 0.0062 and the eigenvalue variance scaled by total variance and the number of variables was 0.35. The eigenvalues, percentages of variance, and cumulative percentages were as follows: PC1: 0.0014, 57.16%, 57.16%; PC2: 0.0004, 16.05%, 73.21%; PC3: 0.0002, 8.31%, 81.52%; PC4: 0.0001, 5.13%, 86.65%; PC5: 0.0001, 4.15%, 90.80%, respectively. As none of the eigenvalues passed the Kaiser rule (eigenvalues > 1), the first three PCs with the highest % variance are displayed in Figure 2A. PCs represent the weights of the partial wraps in the whole warps between all conformations.

Concerning the consensus on the dorsal profile, the extremum of PC1 supported the dorsal profile, with an elevated dorsal line in the hindquarter region, lowered dorsal line in the back region, and slightly elevated dorsal line in the neck and head region with the medial canthus of the eye shifted rostro-dorsally. The extremum of PC2 supported the dorsal profile, with a slightly elevated and elongated dorsal line in the hindquarter region, lowered dorsal line in the back region only in the withers area, lowered dorsal line in the neck region, and elevated dorsal line in the head region, with the medial canthus of the eye shifted slightly caudally. The extremum of PC3 supported the dorsal profile, with a shortened dorsal line in the hindquarter region, elevated and lowered dorsal line in the back region in the area of the lumbar and withers area, respectively, as well as a lowered dorsal line in the neck region and elevated dorsal line in the head region with the medial canthus of the eye shifted rostro-dorsally. The variance in the first three PCs was as follows: PC1 = 57.16%, PC2 = 16.05%, PC3 = 8.31% (Figure 2B). On the general scatter plot of the principal component scores in the PC1 to PC2 orientation, it is easy to see that the scores are partially divided into separate categories of equids. More donkeys represented the dorsal profile supported by PC2, whereas more ponies and horses represented the dorsal profile supported by PC1 (Figure 2C). On detailed scatter plots representing the breed composition, it is easy to see that confidence ellipses separated two breeds of donkeys (Figure 2D) and horses (Figure 2F) and breed one of ponies (Figure 2E). Breeds represented by one, two, or three individuals alternated with breeds represented more numerously. With these sizes of breed groups, no clear separation was evidenced.

The effect of the equid classifier on both the centroid size and shape of the animal’s dorsal profile was reported in Table 1. The centroid size differed between equids globally (*p* < 0.0001), as well as for donkeys versus horses (*p* < 0.0001) and ponies versus horses (*p* < 0.0001), but not for donkeys versus ponies (*p* = 0.171). Similarly, the shapes differed between equids globally (*p* < 0.0001), but also between donkeys and ponies (*p* < 0.0001) and donkeys and horses (*p* < 0.0001), but not ponies and horses (*p* = 0.065).

**Figure 3:**
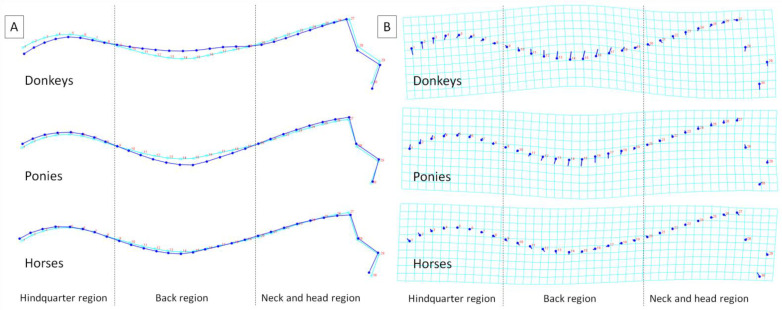
Average observations of the equid dorsal profiles of donkeys, ponies, and horses and represented by (**A**) the wireframe graphs and (**B**) the transformation grids. On the wireframe graphs, light blue landmarks and curves represent the consensus dorsal profile of the animals and dark blue landmarks and curves represent average observations for the subsequent groups. On the transformation grid, dark blue landmarks represent the consensus dorsal profile of the animals, and dark blue lines represent the average observations for subsequent groups. Dashed lines indicate the boundaries between regions.

To better visualize the differences in the dorsal profiles between equids, average observations for consecutive classifiers are displayed in Figure 3. In relation to the consensus dorsal profile, the average donkey was represented by the lowering of the dorsal line in the hindquarter region, the elevation of the dorsal line in the back region, as well as slightly lowering of the dorsal line in the neck and head region with slightly rostro-ventral shift of the medial canthus of the eye. Thus, the deformations of the dorsal line were observed in the hindquarter, back, and neck and head regions. In relation to the consensus dorsal profile, the average pony was represented by the lowering of the dorsal line only in the back region, meaning the deformations of the dorsal line were observed only there. The average horse was represented by a dorsal line similar to the consensus dorsal profile, meaning only minor deformations were observed.

As a summary of the previous results, the distances between the equids’ dorsal profiles are reported in Table 2. Higher distances were noted between donkeys and horses and donkeys and ponies than between ponies and horses.

### 3.2. Comparison of the Equids’ Head Shapes

**Figure 4:**
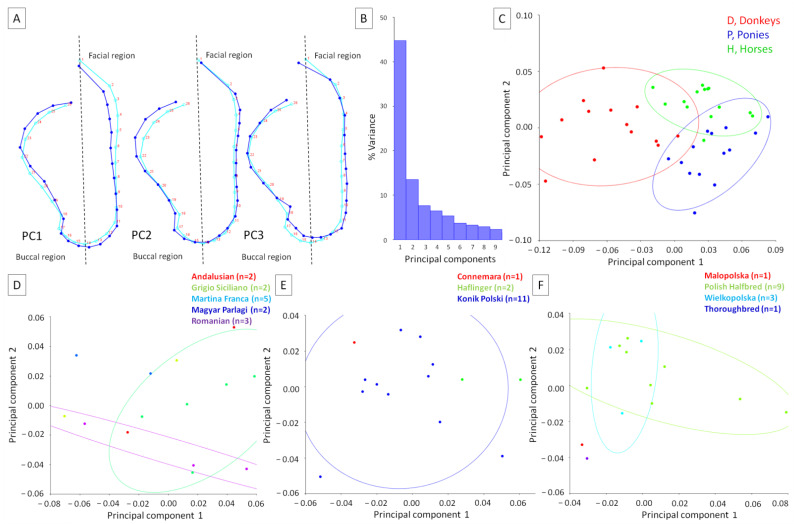
Principal component scores (PCs) of the head shapes of equids, as represented by (**A**) the wireframe graph, (**B**) histogram of variance, and (**C**–**F**) scatter plot of the PCs scores of the equids. On the wireframe graph (**A**), light blue landmarks and curves represent the consensus animal’s head shape. Dark blue landmarks and curves represent the extremum (minimum of the axis) values of PC1, PC2, and PC3, respectively. Dashed lines indicate the boundaries between regions. On the general scatter plot of the PCs scores (**C**), the color for each group was determined based on the classifier variables: D, donkeys; P, ponies; H, horses. On the detailed scatter plots of the PCs scores (**D**–**F**), the color of each breed with the number of individuals (n) is shown for donkeys (**D**), ponies (**E**), and horses (**F**). The confidence ellipses were drawn using a 0.9 probability and with a classifier as a criterion for grouping the observations.

The head shapes of animals were first investigated to identify variations in postures associated with the equid groups. For 26 LDs in 1 dimension, the dataset contained 42 observations, of which 42 were included for analyses. Considering PCA, the total variance was 0.0058 and the variance of the eigenvalues was 0.00000015. The eigenvalue variance scaled by total variance was 0.0045 and the eigenvalue variance scaled by total variance and number of variables was 0.22. The eigenvalues, of variance, and cumulative percentages were as follows: PC1: 0.0025, 44.78%, 44.78%; PC2: 0.00078, 13.46%, 58.24%; PC3: 0.00044, 7.66%, 65.89%; PC4: 0.00037, 6.44%, 72.33%; PC5: 0.00031, 5.32%, 77.65%, respectively. As none of the eigenvalues passed the Kaiser rule (eigenvalues > 1), the first three PCs with the highest % variance are displayed in Figure 4A. PCs represented the weights of the partial wraps in the whole warps between all conformations.

In relation to the consensus on head shape, the extremum of PC1 supported the head shape, with a caudally shifted head line in the upper facial region, lowered head line around the nostrils, and elevated head line in the buccal region. The extremum of PC2 supported the head shape, with a slightly rostrally shifted head line in the facial region and caudally shifted head line in the upper buccal region. The extremum of PC3 supported the head shape, with a rostrally shifted head line in the lower facial region, an elevated head line around the nostrils, and elevated head line in the lower buccal region. The variance of the first three PCs was as follows: PC1 = 44.77%, PC2 = 13.46%, and PC3 = 7.66% (Figure 4B). On the general scatter plot of principal component scores in the PC1 to PC2 orientation, it is easy to see that the scores are partially divided into separate categories of equids. More donkeys represented the head shape supported by PC2, whereas more ponies represented the dorsal profile supported by PC1 (Figure 4C). On detailed scatter plots representing breeds composition, it is easy to see that confidence ellipses separated two breed groups for donkeys (Figure 4D) and horses (Figure 4F) and one group for ponies (Figure 4E). Breeds represented by one, two, or three individuals were alternated with breeds represented more numerously. With these sizes of breeds groups, no clear separation was evidenced.

The effect of the equid classifier on both the centroid size and head shape is reported in Table 3. The centroid sizes differed between equids globally (*p* < 0.0001), as well when donkeys versus horses (*p* < 0.0001) and ponies versus horses (*p* < 0.0001) but not donkeys versus ponies (*p* = 0.125) were compared. Similarly, the shapes differed between equids globally (*p* < 0.0001), between donkeys and ponies (*p* < 0.0001) and donkeys and horses (*p* < 0.0001), but between not ponies and horses (*p* = 0.617).

**Figure 5:**
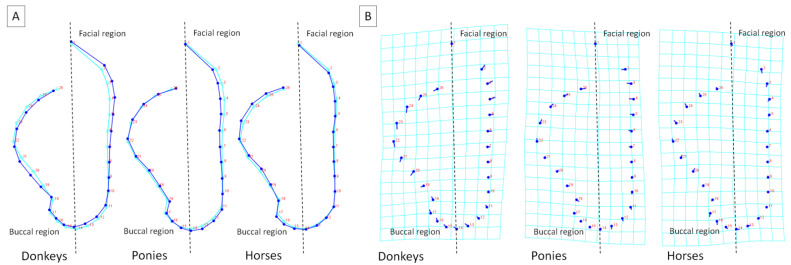
Average observations of the equid head shapes for donkeys, ponies, and horses, represented by (**A**) wireframe graphs and (**B**) transformation grids. On the wireframe graphs, light blue landmarks and curves represent the consensus animal’s head shape and the dark blue landmarks and curves represent the average observations for the subsequent groups. On the transformation grid, the dark blue landmarks represent the consensus animal’s head shape, while dark blue lines represent the average observations for subsequent groups. Dashed lines indicate the boundaries between regions.

To better visualize the differences in head shape between equids, average observations for consecutive classifiers are displayed in Figure 5. In relation to the consensus head shape, the average donkey was represented by the rostrally shifting of the head line in the facial region, the slight elevation of the head line around the nostrils, as well as causal shifting of the head line in the buccal region. Thus, deformations of the head line were observed in the facial and buccal regions. Concerning the consensus head shape, the average pony was represented by the slight caudal shifting of the head line only in the facial region, meaning the deformations of the head line were observed only there. The average horse was represented by the head line being similar to the consensus head shape, meaning only minor deformations were observed.

As a summary of the recent results, the distances between the equids’ head shapes are reported in Table 4. Higher distances were noted between donkeys and horses and donkeys and ponies than between ponies and horses.

## 4. Discussion

Donkeys have certain specific variations that make them different from horses. These include variations in anatomy, behavior, physiology, and susceptibility to diseases [26]. Anatomical differences between horses and donkeys have been reported in the digestive tract [41], spine [42,43], skull [37], and peripheral skeleton [44]. Donkeys differ from horses due to their large ears, short neck, and small feet [26]. Concerning behavioral differences, donkeys, unlike horses, prefer to live alone or in very small groups. Therefore, donkeys do not exhibit the typical herd behaviors manifested by horses [17,26]. Horses are open grassland foragers and social prey animals that prefer flight to defense [45], whereas donkeys may be territorial and display aggression toward other species sharing their space [26]. As mentioned in Section 1, horses and donkeys show differences in their behavioral responses to pain [15,46], in such a way that horses exhibit more obvious signs of pain [32,33,34,35,36] whereas donkeys show more subtle signs [15,28,29,30,31]. Concerning physiological differences, donkeys show more metabolic changes indicative of stress than horses. These differences are evidenced when animals are transported or subjected to mixing at auction houses [47]. Moreover, the nutritional requirements of donkeys are much lower than those of horses, meaning donkeys can survive on much lower quality forage than horses [16,26,48]. However, this adaptive advantage becomes a serious problem when donkeys are housed and fed like horses [16] due to the donkeys’ higher risk of metabolic diseases [16,48,49] and greater risk of obesity compared to horses [16,49].

Despite these indicated differences, donkeys and horses show enough similarities to introduce the methods and scales that were successfully designed and validated for horses as a starting point for the development of specific donkeys scales [15]. Such an introduction was successfully performed for manual [15] but not automatic [27] pain detection approaches in donkey. In the case of manual pain detection, acute head-related pain was best assessed by means of facial-expression-based pain scales similarly in donkeys [15] and horses [34]. However, in the case of automatic pain detection, only some preliminary tests were performed to evaluate the potential of extrapolating a horse-based model to donkey images. However, the difficulties in transferring models to donkey faces were shown [27]. After the application of the trained models on the donkey dataset, Hummel et al. [27] reported a steep drop in results for both pose detection and landmarking. The authors suggested clear differences in the face proportions between horses and donkeys [27], as confirmed during both a recent post-mortem evaluation of equid skulls [37] and in the current evaluation of equid head shapes on live animals in field conditions. The current results, showing that donkey heads differed in shape compared with pony and horse heads, as well as in size compared with horse heads, are in agreement with recent findings [27,37]. Notwithstanding that anatomical morphometry is very detailed and allows for an unambiguous indication of differences in the lengths of individual sections and the values of the calculated indexes [37]. Geometric morphometrics has practical advantages, as it can be performed non-invasively [20,23,24]. Therefore, one may suggest that the “donkeyfying” of the horse head, which Hummel et al. [27] challenged in their automatic pain prediction model for donkeys, should consider enlargements of the facial and buccal regions, reported in this study.

Another method that was designed and validated for horses and translated into donkey welfare evaluations involves a dorsal profile assessment. In horses, the precise geometric morphometric measurements of the dorsal profile were evidenced to certainly reflect horses’ welfare state [24]. In horses, a flat or hollow dorsal profile was related to a compromised welfare state and risk of at least a “depressed” or “abnormal” psychological state [24]. In donkeys, a similar posture was recently reported to be associated with low body condition [20]. In the current study, the impact of low body condition was excluded, as all animals showed BCS values ranging from 2 to 3 in a 5-point scoring system [16]. Therefore, comparing horses, ponies, and donkeys with similar body conditions, one may observe that the shapes of donkeys’ dorsal profiles differed from those in horses and ponies. The donkeys’ dorsal profiles were flatter compared to the more hollow dorsal profiles in horses and ponies, which should be considered in further posture-dependent welfare evaluations. The current results are in agreement with recent findings show that the neck and back shape in donkeys differ from other equines [26,42,43,44]. The shorter lumbar spine in donkeys (five lumbar vertebrae [42]) in relation to horses (six lumbar vertebrae [43]) may explain the interspecific difference in the shape of the dorsal line reported here. However, convictions about the relatively shorter neck in donkeys than in horses [26,42,43] were only partially confirmed in the current study, as the sizes of the dorsal lines differed between donkeys and horses but not between donkeys and ponies.

In the current study, two important limitations should be considered. The first is the numbers of individuals in the analyzed groups. In the previous research, Hummel et al. [27] used 1854 images of horse heads and 531 images of donkey heads, whereas here only 84 images were tested. Moreover, van Dierendonck et al. [15] monitored 264 donkeys, and Sénèque et al. [23,24] measured 85 horses, both numbers being much greater than in this study. On the other hand, Merkies et al. [37] measured 16 donkeys and 14 horses, while we marked 14 donkeys, 14 ponies, and 14 horses. Maher et al. [50] explored 12 donkey heads, whereas here 14 animal heads were in each group. Therefore, following the resource equation method of animal group size calculation [51], the sample size in the current study can be considered adequate. The E value in this study equaled 39 (42 animals − 3 groups), which is more than the acceptable value of 20. The resource equation method was used due to the non-numeric nature of the GM data, lack of standard deviation, and lack of availability of previous findings [51]. Thus, the current study can be considered a pilot study.

Considering the effect of the breeding on equid dorsal profiles and head shapes, breeds represented by one, two, or three individuals alternated with breeds represented more numerously. Therefore, no clear evidence of breed separation was noted, which may be strongly affected by the size of breeds groups. In the previous research, no breed-related differences in dorsal profiles were evidenced using GM for donkeys [20] and horses [23,24]; however, no similar research was reported for head shapes. Knowing the breed-dependent morphological variations in horse body sizes and shapes [52], more GM research, including with donkeys, ponies, and horses of the same breed, is needed to clearly show the effect of the equid breed on the dorsal profile and head shape.

In summary, it can be stated that the GM allows the differentiation of both equid dorsal profiles and head shapes on live animals in field conditions. Nevertheless, further studies are needed to introduce the evidenced interspecies differences into the scales [15,27] and methods [20,23,24,27] transferred from horses to donkeys for welfare assessments. Although the current research was focused on donkeys kept as companions, guards, or recreational animals, it should be emphasized that product consistency, namely in the sourcing and management of donkeys, in intensive production systems is an area of welfare and management that needs additional research [17].

## 5. Conclusions

The use of geometric morphometrics revealed the differences in dorsal profiles and head shapes between the studied equids. A comparison of donkeys, ponies, and horses showed that the dorsal profiles and head shapes differed similarly in size and shape between donkeys and horses but not between donkeys and ponies. Moreover, both dorsal profiles and head sizes differed between ponies and horses, but not head shapes. These tangible differences should be taken into account when adapting scales and methods from horses to donkeys.

## Figures and Tables

**Table 1:** The effect of the equid classifier on both the centroid size and shape of an equid’s dorsal profile determined using Procrustes ANOVA with sum of squares (SS) and mean squares (MS). The significance level was established as *p* < 0.05. The significant effects of the classifier are marked in bold font in the *p*-value column. The bold is used here to separate the headings and the features or data.

**Centroid Size**	**SS**	**MS**	**df**	**F**	***p***
Equid	3,444,784.26	1,722,437.12	2	29.67	**<0.0001**
D vs. P ^1^	169,763.49	169,763.48	1	1.99	0.171
D vs. H ^2^	3,144,525.96	3,144,525.92	1	99.42	**<0.0001**
P vs. H ^3^	148,290.19	57,035.77	1	32.49	**<0.0001**
**Shape**	**SS**	**MS**	**df**	**F**	***p***
Equid	0.040	0.0004	112	12.20	**<0.0001**
D vs. P ^1^	0.032	0.0005	56	19.97	**<0.0001**
D vs. H ^2^	0.022	0.0004	56	12.14	**<0.0001**
P vs. H ^3^	0.009	0.00005	168	3.92	0.065

^1^ Comparison repeated after combination of donkey data set (D) and pony data set (P). ^2^ Comparison repeated after combination of donkey data set (D) and horse data set (H). ^3^ Comparison repeated after combination of pony data set (P) and horse data set (H).

**Table 2:** Mahalanobis distances (MD) and Procrustes distances (PD) among the equids’ dorsal profile categories (donkeys, ponies, and horses).

		Donkeys	Ponies
Ponies	MD	18.65	
	PD	0.068	
Horses	MD	22.16	9.53
	PD	0.056	0.027

**Table 3:** The effects of the equid classifier on both the centroid size and shape of an equid’s head determined using the Procrustes ANOVA with sum of squares (SS) and mean squares (MS). The significance level was established as *p* < 0.05. Significant effects of the classifier are marked in bold font in the *p*-value column. The bold is used here to separate the heading and features or data.

**Centroid Size**	**SS**	**MS**	**df**	**F**	***p***
Equid	429,195.33	214,597.67	2	30.62	**<0.0001**
D vs. P ^1^	26,212.04	26,212.04	1	2.51	0.125
D vs. H ^2^	397,797.56	397,797.56	1	89.86	**<0.0001**
P vs. H ^3^	219,783.39	219,783.39	1	35.62	**<0.0001**
**Shape**	**SS**	**MS**	**df**	**F**	***p***
Equid	0.092	0.0010	96	12.45	<0.0001
D vs. P ^1^	0.065	0.0014	48	16.34	<0.0001
D vs. H ^2^	0.056	0.0011	48	13.73	<0.0001
P vs. H ^3^	0.002	0.00003	48	0.93	0.617

^1^ Comparison repeated after combination of donkey data set (D) and pony data set (P). ^2^ Comparison repeated after combination of donkey data set (D) and horse data set (H). ^3^ Comparison repeated after combination of pony data set (P) and horse data set (H).

**Table 4:** Mahalanobis distances (MD) and Procrustes distances (PD) among the equid head shape categories (donkeys, ponies, and horses).

		Donkeys	Ponies
Ponies	MD	15.44	
	PD	0.096	
Horses	MD	17.77	7.93
	PD	0.090	0.050

## Data Availability

The data presented in this study are available on request from the corresponding author.
